# Validation and Modification of a Prediction Model for Acute Cardiac Events in Patients With Breast Cancer Treated With Radiotherapy Based on Three-Dimensional Dose Distributions to Cardiac Substructures

**DOI:** 10.1200/JCO.2016.69.8480

**Published:** 2017-01-17

**Authors:** Veerle A.B. van den Bogaard, Bastiaan D.P. Ta, Arjen van der Schaaf, Angelique B. Bouma, Astrid M.H. Middag, Enja J. Bantema-Joppe, Lisanne V. van Dijk, Femke B.J. van Dijk-Peters, Laurens A.W. Marteijn, Gertruida H. de Bock, Johannes G.M. Burgerhof, Jourik A. Gietema, Johannes A. Langendijk, John H. Maduro, Anne P.G Crijns

**Affiliations:** All authors: University Medical Center Groningen, University of Groningen, Groningen, the Netherlands

## Abstract

**Purpose:**

A relationship between mean heart dose (MHD) and acute coronary event (ACE) rate was reported in a study of patients with breast cancer (BC). The main objective of our cohort study was to validate this relationship and investigate if other dose-distribution parameters are better predictors for ACEs than MHD.

**Patients and Methods:**

The cohort consisted of 910 consecutive female patients with BC treated with radiotherapy (RT) after breast-conserving surgery. The primary end point was cumulative incidence of ACEs within 9 years of follow-up. Both MHD and various dose-distribution parameters of the cardiac substructures were collected from three-dimensional computed tomography planning data.

**Results:**

The median MHD was 2.37 Gy (range, 0.51 to 15.25 Gy). The median follow-up time was 7.6 years (range, 0.1 to 10.1 years), during which 30 patients experienced an ACE. The cumulative incidence of ACE increased by 16.5% per Gy (95% CI, 0.6 to 35.0; *P* = .042). Analysis showed that the volume of the left ventricle receiving 5 Gy (LV-V5) was the most important prognostic dose-volume parameter. The most optimal multivariable normal tissue complication probability model for ACEs consisted of LV-V5, age, and weighted ACE risk score per patient (c-statistic, 0.83; 95% CI, 0.75 to 0.91).

**Conclusion:**

A significant dose-effect relationship was found for ACEs within 9 years after RT. Using MHD, the relative increase per Gy was similar to that reported in the previous study. In addition, LV-V5 seemed to be a better predictor for ACEs than MHD. This study confirms the importance of reducing exposure of the heart to radiation to avoid excess risk of ACEs after radiotherapy for BC.

## INTRODUCTION

The number of breast cancer (BC) survivors is increasing as a result of rising incidence, earlier diagnosis, and better treatment results.^1,2^ Although adjuvant radiotherapy (RT) after surgery for BC improves locoregional control and overall survival, incidental exposure of the heart to radiation increases the risk of RT-induced cardiac toxicity.^3-5^ Consequently, the prevalence of BC survivors at risk for long-term RT-induced cardiac toxicity is increasing and may have a significant impact on health-related quality of life.

Darby et al^6^ demonstrated a dose-effect relationship based on the mean heart dose (MHD) to the whole heart. They found a relative increase of 7.4% per Gy of MHD in the rate of major acute coronary events (ACEs) for the entire follow-up period. Confining the analysis to the first 9 years after radiation exposure, a relative increase of approximately 16% per Gy was found. However, the study had some limitations: its design (case-control study), use of outdated RT technologies, and use of reconstructed MHDs derived from two-dimensional data.

Therefore, the first aim of our study was to validate the findings of Darby et al^6^ with an independent cohort of consecutive patients with BC based on individual three-dimensional (3D) dose distributions derived from computed tomography (CT) planning scans. The second aim of this cohort study was to investigate whether other dose-distribution parameters could better predict the excess risk of ACEs after RT in individual patients with BC compared with MHD.

## PATIENTS AND METHODS

### Study Population

**Fig A1.**
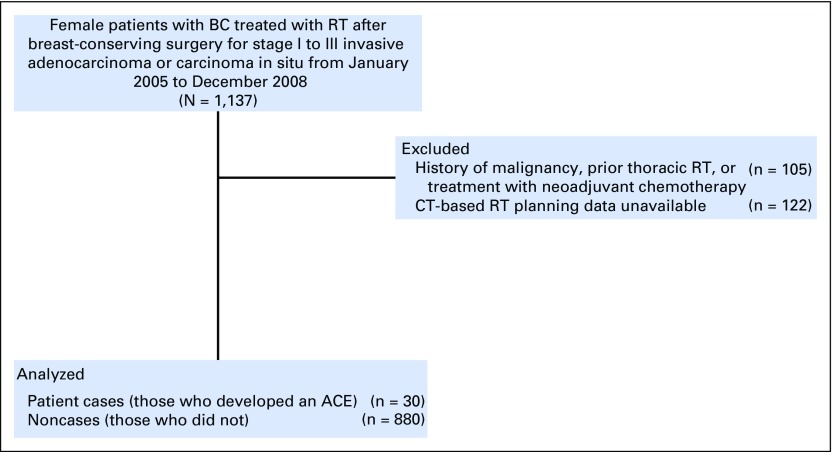
Study population flowchart. ACE, acute coronary event; BC, breast cancer; CT, computed tomography; RT, radiotherapy.

This study population was composed of a consecutive series of female patients with BC treated with RT after breast-conserving surgery for stage I to III invasive adenocarcinoma or carcinoma in situ from January 2005 to December 2008 in our hospital (Appendix Fig A1, online only). Patients with BC were eligible for inclusion only if CT-based RT planning data were available. Patients were excluded if they had a history of other malignancies or had received prior RT or treatment with neoadjuvant chemotherapy. The primary end point was an ACE, defined as a diagnosis of myocardial infarction (International Classification of Diseases, 10th Revision, codes 121 to 124), coronary revascularization, or death resulting from ischemic heart disease (codes 120 to 125) after completion of treatment. Pretreatment risk factors for ACEs that were taken into account included history of ischemic heart disease, any other cardiac disease, hypertension, chronic obstructive pulmonary disease, pulmonary embolism, diabetes, current smoker status, and body mass index ≥ 30 kg/m^2^. Both the end point and pretreatment risk factors were similar to those defined by Darby et al.^6^

### Data Collection

Patient characteristics, treatment plans, follow-up data, and information on cardiac risk factors and cardiac end points were retrospectively extracted from patient records of the Department of Radiation Oncology (University Medical Center Groningen, University of Groningen, Groningen, the Netherlands). Incomplete patient data were supplemented with information derived from general practitioners’ (GPs’) records. To this end, surviving patients were informed about the study by letter and asked for their written informed consent. GPs of deceased patients were allowed to provide relevant information directly, because GPs have legal governance over deceased patients’ records in the Netherlands. The aforementioned procedure was approved by the medical ethical committee of the University Medical Center Groningen.

### Data Definitions

The baseline date was defined as the first day of breast irradiation. Patient event times were censored in cases where a new radiation treatment was delivered in the follow-up period, in cases of death, or at the end of follow-up time. The follow-up interval was defined as the time between baseline and censoring date or date of event. Patient information was collected until the last known date of medical follow-up or last known information obtained from the GP.

### Radiation Dosimetry

Irradiation of the breast for all patients was performed with 3D conformal RT using CT-based planning, as described previously.^7^ All treatment plans were calculated using heterogeneity corrections. Beam configuration comprised tangential fields and additional beams for optimization of planning target volume coverage, as well as for minimization of the dose to the heart, lungs, and contralateral breast. A dose of 50.4 Gy was prescribed for the whole breast in 28 fractions, with a simultaneous integrated boost dose of 14 or 16.8 Gy in the same 28 fractions, depending on pathologic risk factors. The heart and its substructures, including the left ventricle (LV), left atrium, right ventricle, and right atrium, were recontoured with a multiatlas automatic segmentation tool of the heart developed in house based on the atlas by Feng et al^8^ (Mirada RTx [version 1.6]; Mirada Medical, Oxford, United Kingdom).^9^ Automatic segmentation reduces interobserver variability in contouring organs at risk and therefore generates more consistent data to create normal tissue complication probability (NTCP) models.^10,11^ With the delineated volumes, it was possible to calculate the exact planned radiation dose to the different volumes. This so-called dose-volume histogram showed the relationship between the dose in Gy to the volume percentage of the structure of interest.^12,13^ With the dose of the individual patients, the dose-effect relationship could be calculated independently of RT technique or treatment volume. Finally, the planned dose-distribution parameters for the whole heart and its substructures were extracted from our treatment planning system (Pinnacle [version 9.1]; Philips Radiation Oncology, Fitchburg, WI).

### Statistical Analysis

The cumulative incidence of ACEs was analyzed using the Kaplan-Meier method. To validate the model of Darby et al,^6^ a multivariable Cox regression analysis was used, including the same risk factors and end point. Model performance was tested for calibration using the Hosmer-Lemeshow (HL) test, and discrimination was tested using the c-statistic.

The most relevant dose-distribution parameters for the different cardiac substructures were identified by comparing the mean dose-distribution parameters of patient cases (patients who experienced an ACE) with noncases (patients who did not). To this end, we calculated the mean V(x) in bins of 5 Gy for both patient cases and noncases, where V(x) refers to the relative volume (in percentage) of the heart or cardiac substructure that received x Gy. Differences between the two groups regarding all mean dose-distribution parameters were tested with a *t* test or Wilcoxon rank sum test whenever appropriate. The dichotomous variable (no risk factor *v* one or more risk factors) was replaced by a weighted ACE risk score per patient. To this end, we first investigated which risk factors were significantly associated with the incidence of ACEs by using univariable Cox regression analysis and then performed a multivariable analysis taking into account only the significant cardiac risk factors. For the risk factors that were significantly associated with ACEs in the multivariable analysis, the regression coefficients were calculated and used for the weighted sum of the risk factor(s) per patient. In correspondence with Darby et al,^6^ age was entered into the model as well. Because the number of events was limited, we decided not to add more than these three factors to the model to prevent overfitting.^14,15^ For internal validation and adjustment for possible internal optimism for both the c-statistics and some estimators, bootstrapping was performed by using 1,000 random subsets. Model performance was tested for calibration using the HL test. Finally, the excess risk of an ACE resulting from RT was calculated via the individual patient risk based on the model minus the individual patient risk assuming the LV receiving 5 Gy (LV-V5) received 0%. Calculations were performed SPSS software (version 22; SPSS, Chicago, IL).

## RESULTS

### Patient Characteristics

A total of 910 patients were included in this study. The characteristics of these patients are summarized in Table 1. The median age of all patients was 59 years (range, 26 to 84 years). At baseline, more than half of the patients had one or more risk factors for ACEs. The median follow-up time was 7.6 years (range, 0.1 to 10.1 years).

**Table 1.**
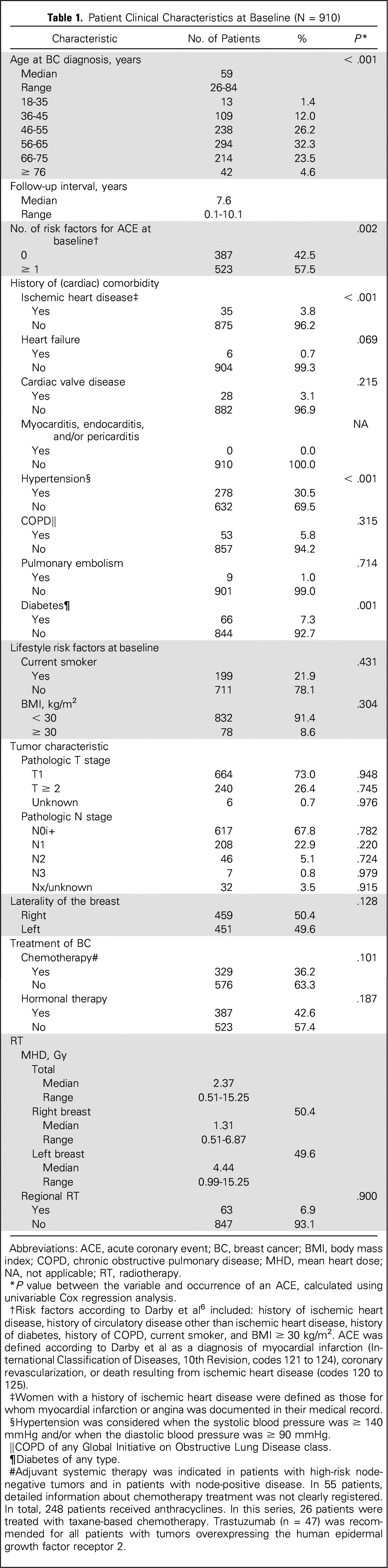
Patient Clinical Characteristics at Baseline (N = 910)

**Fig A2.**
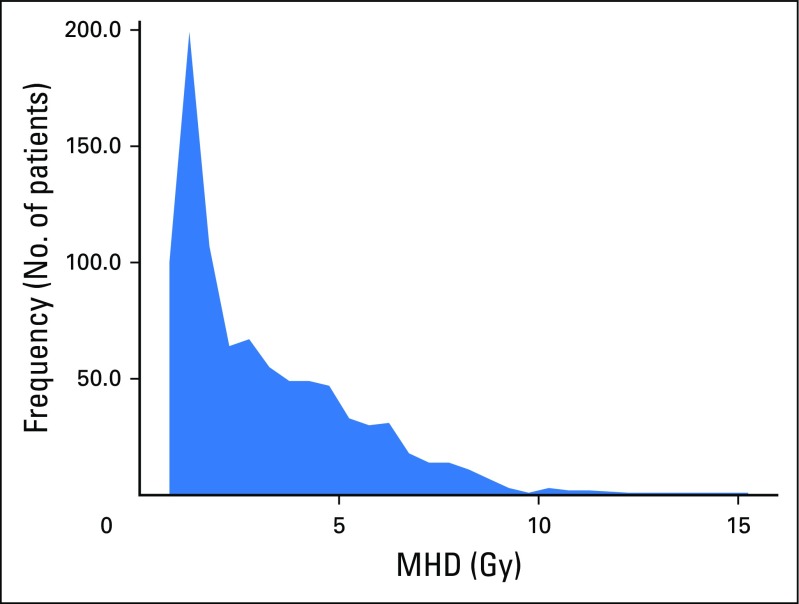
Distribution of mean heart dose (MHD) for the entire population (N = 910; median MHD, 2.37; standard deviation, 2.26; range, 0.51 to 15.25).

**Fig A3.**
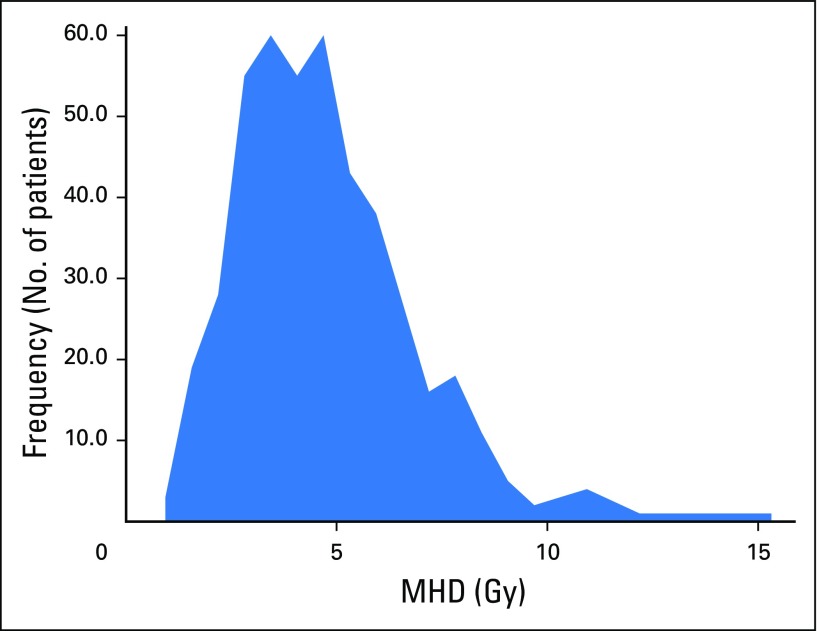
Distribution of mean heart dose (MHD) for left-sided breast cancer (n = 451; median MHD, 4.44; standard deviation, 2.12; range, 0.99 to 15.25).

**Fig A4.**
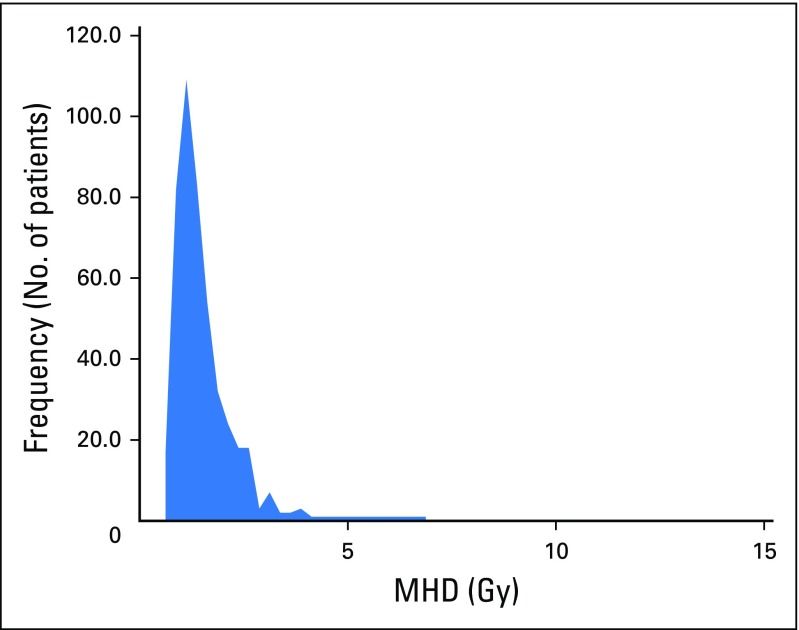
Distribution of mean heart dose (MHD) for right-sided breast cancer (n = 459; median MHD, 1.31; standard deviation, 0.72; range, 0.51 to 6.87).

**Fig A5.**
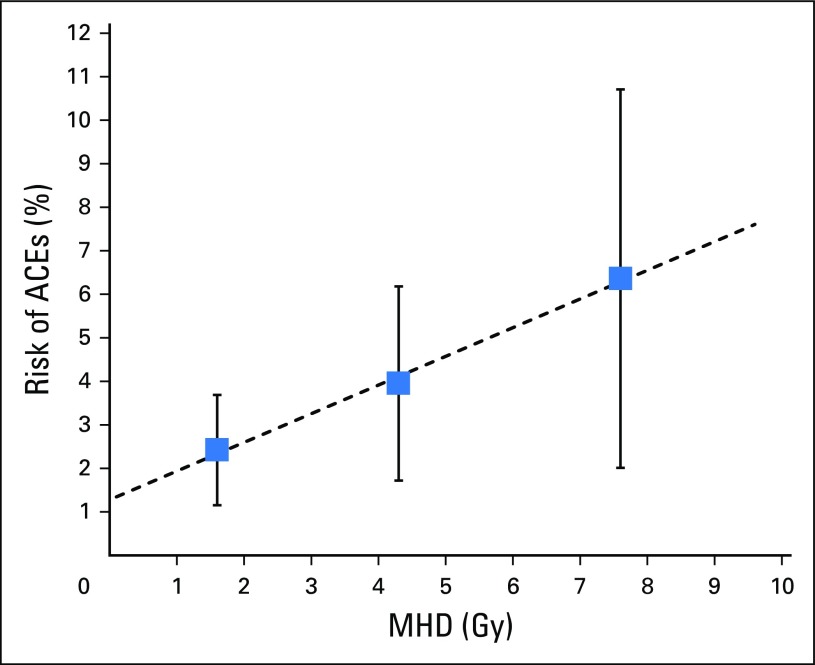
Relationship between mean heart dose (MHD) and percentage of acute coronary events (ACEs) based on univariable analysis (ie, not corrected for age or presence of cardiovascular risk factors). The linear trend line crosses the y-axis, indicating ACEs not related to radiotherapy. Vertical bars indicate 95% CIs.

**Fig A6.**
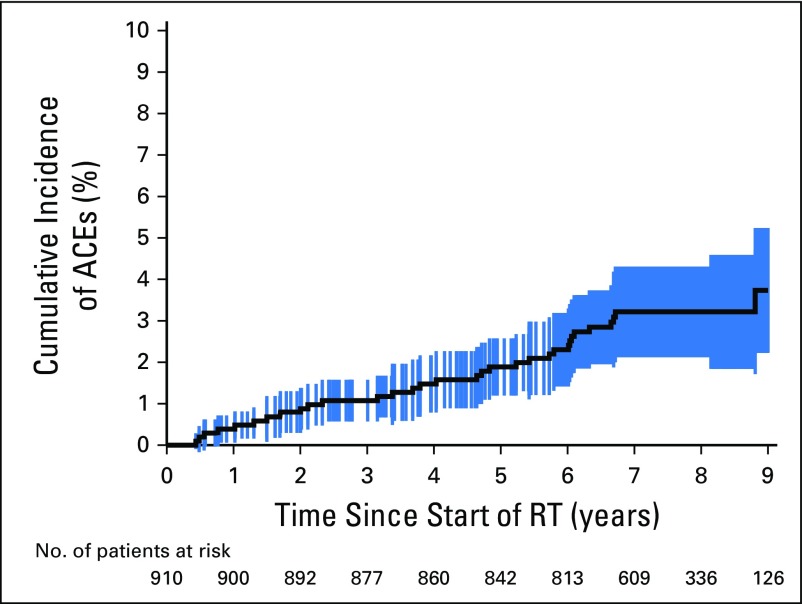
Cumulative incidence of acute coronary events (ACEs) in the entire population; vertical bars indicate 95% CIs. RT, radiotherapy.

More detailed information about the distribution of MHD and the univariable analysis between MHD and the end point ACE is provided in Appendix Table A1 (online only), Appendix Figures A2 to A4 (online only), and Appendix Figure A5 (online only), and information about patients experiencing an event is listed in Appendix Table A2 (online only). In total, 30 patients (3.3%) developed an ACE during follow-up, 10 of whom died as a result of ischemic heart disease. In the first 5 years, 17 patients were diagnosed with ACEs. The 5- and 9-year cumulative incidences of ACEs were 1.9% (95% CI, 0.9% to 2.9%) and 3.9% (95% CI, 2.3% to 5.5%), respectively (Appendix Fig A6, online only).

### Validation

To validate the model of Darby et al,^6^ a multivariable Cox regression model was created using the same prognostic factors (ie, age, MHD, and presence of pretreatment risk factors for ACEs, categorized as either none or one or more at baseline). The cumulative incidence of ACEs increased by 16.5% per Gy (*P* = .042) within 9 years of RT (Table 2).

**Table 2.**
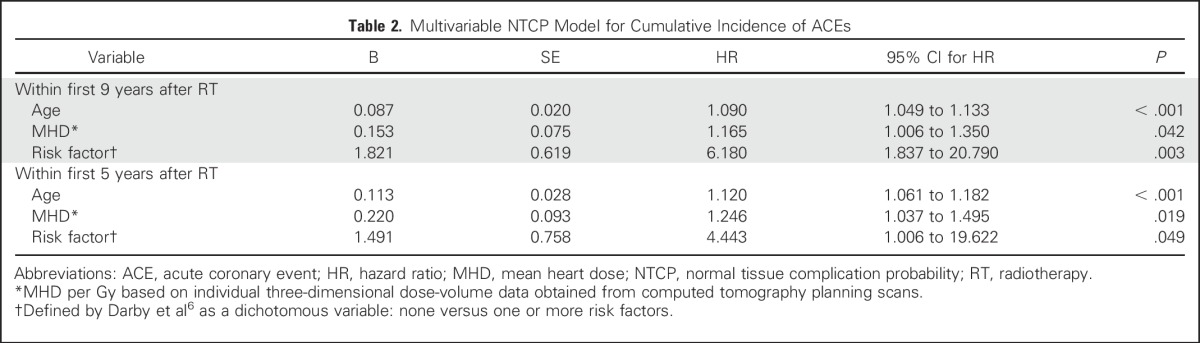
Multivariable NTCP Model for Cumulative Incidence of ACEs

On the basis of this model, the 9-year excess cumulative risk (CER_9y_) can be calculated using the following equations:

1. The linear predictor LP_MHD-model_ = (0.153 × MHD) + (0.087 × AGE) + (1.821 × RISK), in which MHD = mean heart dose in Gy, AGE = age in years, and RISK = 0 when no risk factors for ACEs are present at baseline and RISK = 1 if one or more risk factors at baseline are present.
2. The cumulative incidence for each individual patient at 9 years (CI_9y_) can then be calculated using the following equation: CI_9y_ = 1 – [EXP(−0.000025 × EXP(LP_MHD-model_))].
3. The 9-year excess cumulative risk (CER_9y_) can then be calculated by using Equation 2 minus the CI_9y_ assuming an MHD of 0 Gy (CI_9y-0Gy_): CER_9y_ = CI_9y_ – CI_9y-0Gy_.

The HL test showed no significant difference between expected and observed rates of ACEs (*P* = .406), indicating good calibration. Model discrimination was good, with a c-statistic of 0.79 (95% CI, 0.71 to 0.87). The mean predicted CI_9y_ for the entire population was 4.0%, which was in agreement with the CI_9y_ actually observed: 3.9%.

To get an impression of the early risk of ACEs, a model for the first 5 years after RT (Table 2) was tested separately. Using the same risk factors and end point as those of Darby et al,^6^ an increase of 24.6% in the rate of ACEs per Gy of MHD was found for the complete follow-up period of 5 years.

### Model Optimization

**Fig 1.**
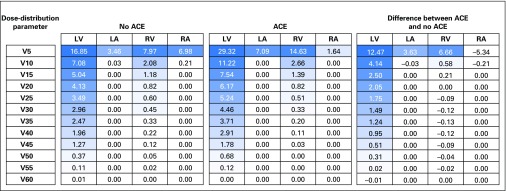
Comparison of the mean dose distribution parameters of patient cases (patients who experienced an acute coronary event [ACE]) and noncases (those who did not) and calculation of the differences. NOTE. All data are given as the relative volumes (%) of the cardiac substructures that received (x) Gy or more in bins of 5 Gy. LA, left atrium; LV, left ventricle; RA, right atrium; RV, right ventricle.

To identify the most relevant dose-distribution parameters, we compared the mean dose parameters of the patient cases (patients who experienced an ACE) with noncases (patients who did not). Figure 1 shows the differences between the mean dose-distribution parameters per cardiac substructure that were tested for significance. The largest difference was found for LV-V5. In the univariable Cox regression analysis, summarized in Table 3, LV-V5 was significantly associated with the cumulative incidence of ACEs, with a hazard ratio of 1.016 (95% CI, 1.002 to 1.030; *P* = .016). Because of this strong association, we chose to include LV-V5 in the model. Replacement of MHD with LV-V5 resulted in an improvement of the c-statistic of the NTCP model to 0.80 (95% CI, 0.72 to 0.88). We also tested the relationship of the maximum dose to the heart with the cumulative incidence of ACEs using a univariable Cox regression analysis and found it was not significantly associated with ACEs (data not shown).

**Table 3.**
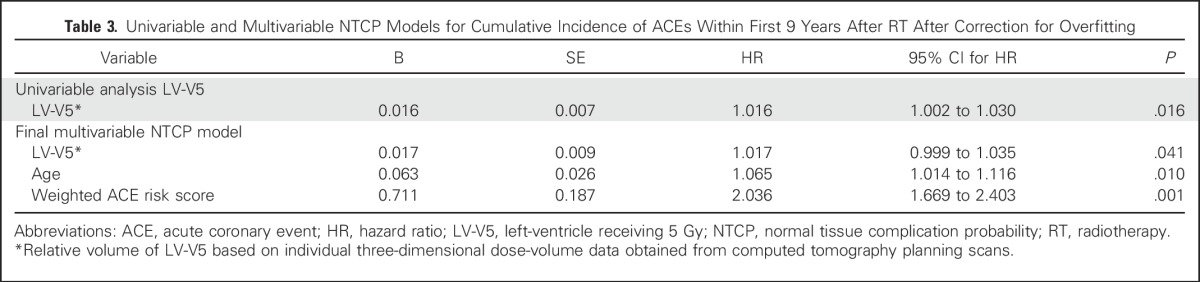
Univariable and Multivariable NTCP Models for Cumulative Incidence of ACEs Within First 9 Years After RT After Correction for Overfitting

To further optimize the NTCP model based on LV-V5, the dichotomous variable (no risk factor *v* one or more risk factors) was replaced with a weighted ACE risk score per patient. Because there were only 30 events, LV-V5, age, and weighted ACE risk score per patient based on the regression coefficient of the significant risk factors for ACEs (0.8 for diabetes, 1.4 for hypertension, and 1.8 for history of ischemic cardiac events) were entered into the multivariable model. The final multivariable NTCP model summarized in Table 3 is corrected for optimism.

On the basis of this model, the 9-year excess cumulative risk (CER_9y_) can be calculated using the following equations:

1. The linear predictor LP_LV-V5-model_ = (0.017 × LV-V5) + (0.063 × AGE) + (0.711 × RISKSCORE), in which LV-V5 = LV-V5 in %, AGE = age in years, and RISKSCORE = weighted ACE risk score (0 for no risk factors; add 0.8 in case of diabetes, add 1.4 in case of hypertension, and add 1.8 in case of ischemic cardiac events before RT).
2. The cumulative incidence for each individual patient at 9 years (CI_9y_) can then be calculated using the following equation: CI_9y_ = 1 – [EXP(−0.000223 × EXP(LP_LV-V5-model_))].
3. The 9-year excess cumulative risk (CER_9y_) can then be calculated by using Equation 2 minus the CI_9y_ assuming an LV-V5 of 0% (CI_9y-0%_): CER_9y_ = CI_9y_ – CI_9y-0Gy_.

The mean predicted CI_9y_ for the entire population was 3.5%, which was in agreement with the CI_9y_ actually observed: 3.9%. This modified model showed good agreement between expected and observed rates of ACEs (HL test *P* = .380). Discrimination of the final model in terms of the c-statistic showed good results at 0.83 (95% CI, 0.75 to 0.91), which was significantly better than that in the MHD model (*P* = .042).

**Fig 2.**
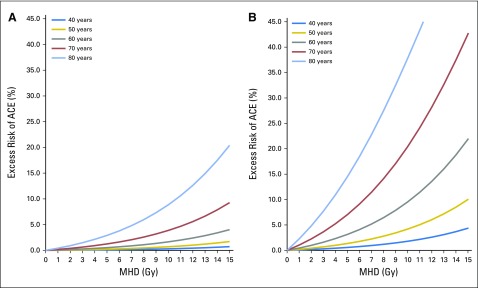
Excess risk of an acute coronary event (ACE) depending on the mean heart dose (MHD) in volume percentage calculated per age category and (A) absence or (B) presence of cardiac risk factors.

**Fig 3.**
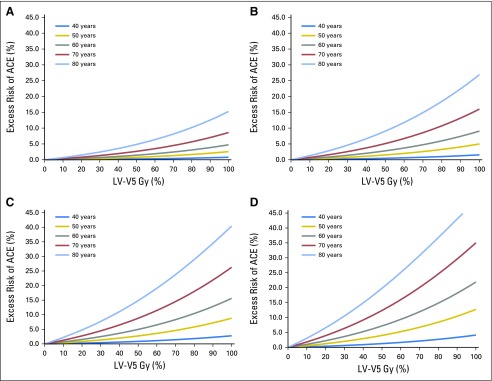
Excess risk of an acute coronary event (ACE) depending on the mean V5 of the left ventricle (LV-V5) in volume percentage calculated per age category and risk factor: (A) no cardiac risk factors, (B) diabetes, (C) hypertension, and (D) ischemic cardiac event. For example, a patient age 70 years with an LV-V5 of 50% and no cardiac risk factors has an excess risk of 2.52% of developing an ACE within 9 years after radiotherapy. If the same patient had a history of ischemic heart disease, with a similar value for LV-V5, the excess risk would increase to 8.42%.

The excess cumulative risk related to RT was 1.13% within 9 years of follow-up, indicating that approximately 10 patients in this BC cohort experienced an ACE that could be attributed to RT. The excess risk for the occurrence of an ACE, depending on the mean dose, is shown in Figure 2 and based on the LV-V5 in Figure 3.

## DISCUSSION

To our knowledge, this is the first study to validate the model published by Darby et al^6^ in an independent cohort using individual 3D CT planning data. Using exactly the same risk factors and end point as Darby et al, we found an increase of 16.5% (95% CI, 0.6 to 35.0) in the cumulative incidence of ACEs per Gy of radiation to the whole heart in the first 9 years after treatment. These results are consistent with the hazard ratios of 16.3% increase per Gy, as observed by Darby et al in the first 4 years of follow-up, and 15.5% increase in the next 5 to 9 years after RT. Furthermore, our study suggests that the NTCP model for ACEs could be improved by using LV-V5 instead of MHD. Model performance showed good results in terms of calibration and discrimination.

An NTCP model is a term generally used in radiation oncology, which refers to any prediction model describing the relationship between 3D dose-distribution parameters of normal tissues and a complication end point. In radiotherapy, NTCP models are generally used to estimate the risks of adverse effects, as well as to optimize dose distributions for individual patients by minimizing the most relevant dose metrics derived from NTCP models.^16^ To enhance the clinical utility of prediction models, it is highly recommended that the performance of the model be evaluated in an independent data set.^17^ Despite differences with regard to study design (case-control *v* cohort study), irradiation technique, estimated dose distributions (reconstructed MHD *v* 3D planning CT based), timeframe, and nationality, the results found in our study are in line with those reported by Darby et al.^6^ Therefore, the model summarized in Table 2 can be considered as a TRIPOD (Transparent Reporting of a Multivariable Prediction Model for Individual Prognosis or Diagnosis) type IV prediction model, the performance of which has been evaluated in an independent data set.^17^ The results of case-control studies, as reported by Darby et al, provide only relative risk against baseline risk, which requires other prediction models to assess these baseline risks. Because our multivariable externally validated model (Table 2) was based on a cohort study, it allows for a direct risk estimation of ACEs for individual patients with BC. However, because we were not able to externally validate the LV-V5 model, this model should be regarded as TRIPOD type Ib, which requires external validation first before it can be used in routine clinical practice.

Our dose-distribution analysis (Fig 1) showed that the LV received the highest dose of all cardiac structures, which is mainly because of the anatomic location of the LV in relation to the breasts and treatment technique, which may increase statistical power. The analysis comparing the dose-distribution parameters between patient cases and noncases also revealed large differences, even for lower dose levels (eg, LV-V2 to -V4; data not shown). LV-V5 was eventually chosen because this dose-distribution parameter has been widely used in many other recent reports.^18-22^

As shown in a recent study, heart doses from RT for BC vary widely, even among seemingly similar regimens.^23^ Therefore, we chose to use an automatic delineation tool to exclude interobserver variability.^8,24^ Furthermore, we used individual dose-volume data, which account for differences in anatomy and treatment volume.

It has long been assumed that the clinical events of incidental cardiac irradiation occur after more than 10 years.^25-29^ One of the biologic mechanisms leading to radiation-induced ACEs is accelerated atherosclerosis.^30-32^ However, in our analysis, a dose-effect relationship was found for events occurring within the first 5 years after radiation exposure. This early risk is consistent with that reported by Darby et al^6^ and that seen in other research in patients with Hodgkin lymphoma.^33^ However, other studies found only a small effect in 6 to 10 years after treatment, when the internal mammary nodes were not treated.^34,35^ When these nodes were treated, the occurrence of cardiac damage was found within 5 years.^36^ Given these results, and setting aside the relatively slowly progressing phenomenon of atherosclerosis, other biologic mechanisms are most likely responsible for the relatively early cardiac events occurring after RT (eg, microvascular damage, impairment in myocardial perfusion and/or fatty acid metabolism, and many more).^37-41^ Studies investigating these underlying mechanisms for early RT-induced cardiac damage using modern imaging techniques are currently under way.^42^

A limitation of our study is the relatively small number of ACEs. Because 3D conformal RT at our hospital was clinically introduced in 2005, the follow-up time was relatively short.

To prevent overfitting by using too many candidate variables in relation to the number of events, we included only two other prognostic factors, besides the dose-distribution parameter: clinical risk factors for ACEs and age, based on the fact that these are considered the most important predictors for ACEs.^43^ Consequently, the effects of other potential confounders could not be taken into account, such as the addition of systemic agents that could also cause cardiac toxicity.^44,45^

In conclusion, the MHD-based NTCP model for ACEs has been independently validated using 3D dose-distribution data among patients with BC treated with postoperative RT. Radiation dose to the heart is an important risk factor for ACEs in BC survivors. Model performance was significantly improved by replacing MHD with LV-V5 and using the weighted ACE risk score, but this optimized model requires further external validation in an independent data set.

## Figures and Tables

**Table A1.**
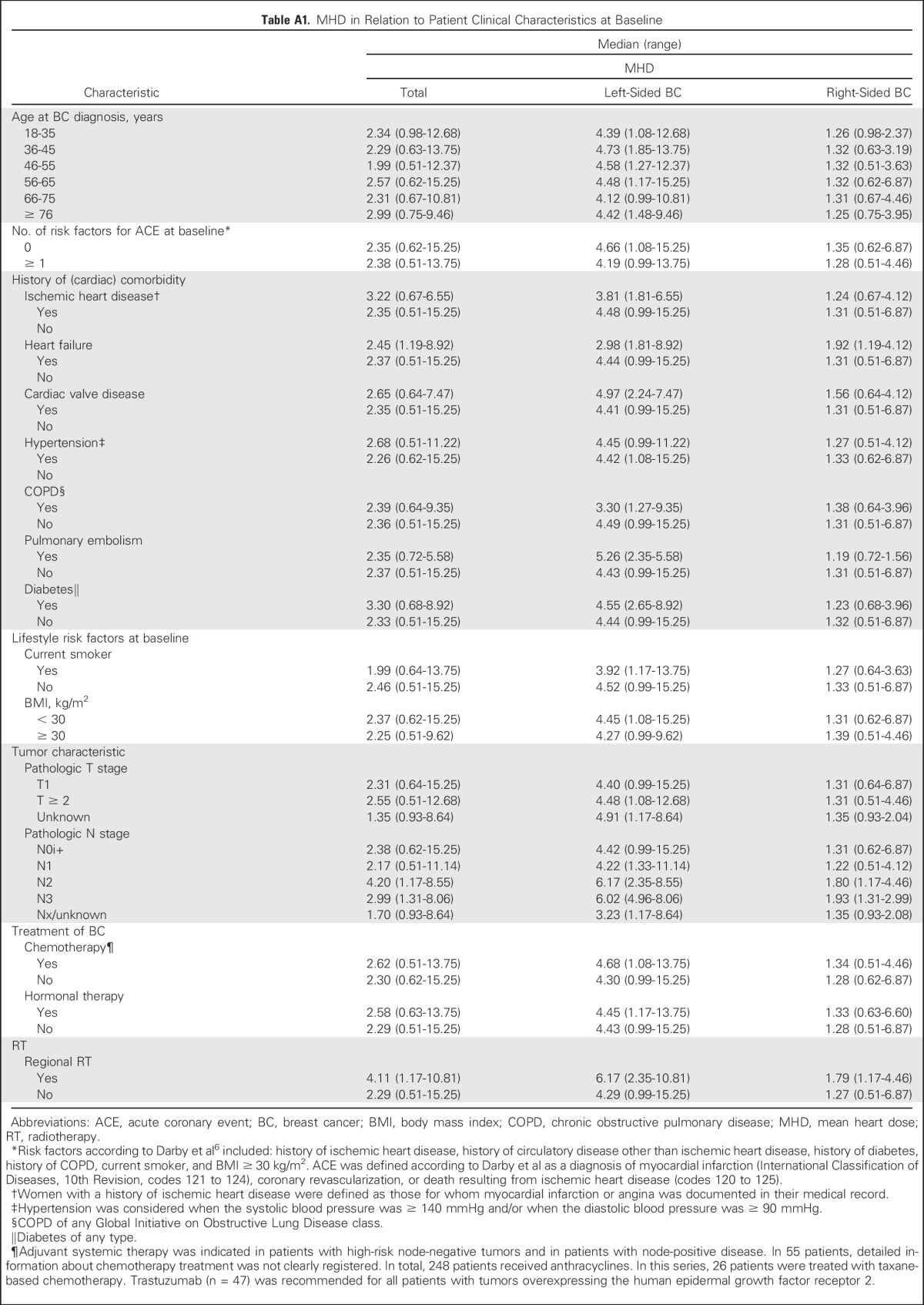
MHD in Relation to Patient Clinical Characteristics at Baseline

**Table A2.**
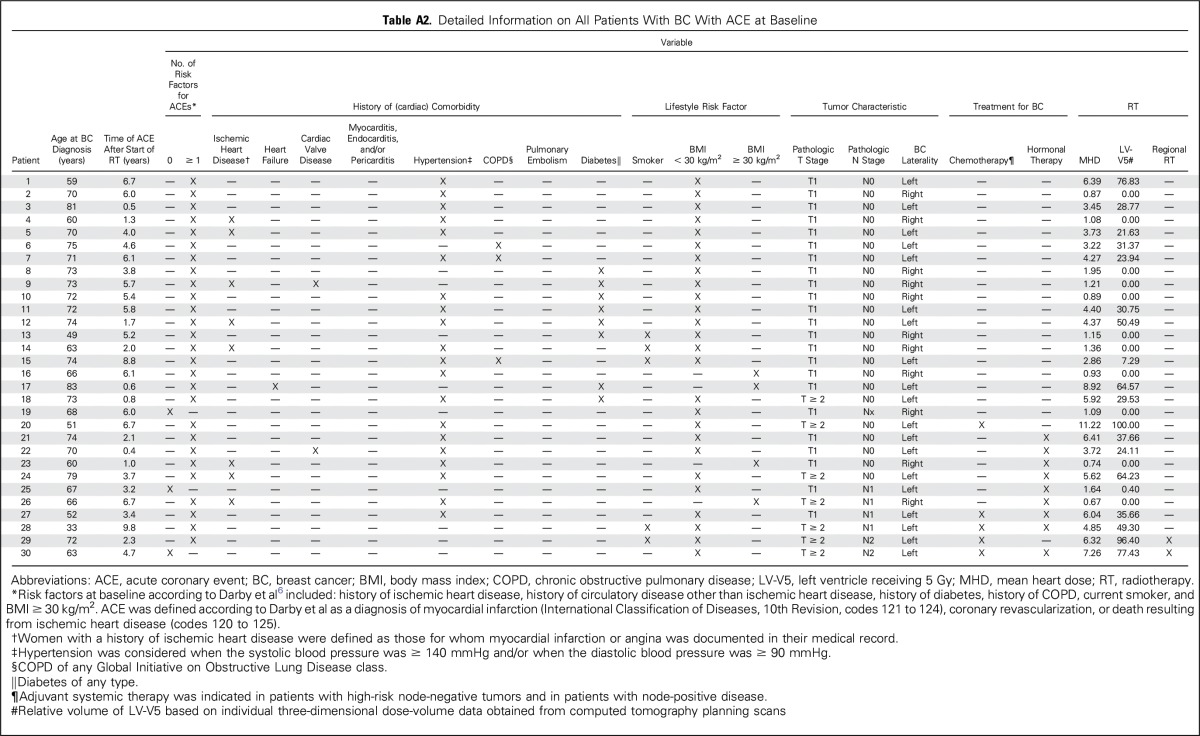
Detailed Information on All Patients With BC With ACE at Baseline
